# The media and the early dissemination of information on medicines for COVID-19: current scenario in Pakistan

**DOI:** 10.31744/einstein_journal/2021CE6354

**Published:** 2021-04-08

**Authors:** Marcos Roberto Tovani-Palone, Sajjad Ali

**Affiliations:** 1 Universidade de São Paulo Faculdade de Medicina de Ribeirão Preto Ribeirão PretoSP Brazil Faculdade de Medicina de Ribeirão Preto, Universidade de São Paulo, Ribeirão Preto, SP, Brazil.; 2 Ziauddin University Faculty of Medicine Karachi Pakistan Faculty of Medicine, Ziauddin University, Karachi, Pakistan.

Dear Editor,

We are currently experiencing an unprecedented pandemic in the modern times.^(^^1^^)^ In this context, the media may have a great influence on the population.^(^^2^^)^ In light of this, there is a concern regarding the dissemination of information on possible preventive and curative benefits of some medicines for coronavirus disease 2019 (COVID-19), such as hydroxychloroquine, which have been early disseminated by various media and governments.^(^^3^^)^ This issue may, in turn, result in negative impacts on the health of the population.^(^^4^^)^ Here, we briefly present the current scenario in Pakistan on the use of early disseminated medicines for COVID-19.

## SCENARIO IN PAKISTAN

The COVID-19 pandemic has affected almost every country in the world, including the region of Pakistan. Despite of this fact, the population of the country is becoming less vigilant, a situation particularly clear due to the gradual easing of the lockdown restrictions, which have had a great impact on the increase in the number of confirmed cases of the disease.^(^^5^^)^ It seems that so far people have not perceived that we are experiencing a serious pandemic, which requires urgency measures and discipline. Moreover, some unproven effects of natural herbs have been disseminated through the media and websites as possible treatments for COVID-19, and many of the residents have now chosen to use such herbs. The most popular of them all is *Cassia angustifolia Vahl*., known as *Senna Makki*,^(^^6^^)^ which is often used as a laxative.^(^^7^^)^ Nevertheless, this herb has been suggested to be life-threatening instead of a cure for the disease. This is because the use of *Senna Makki* tea can result in electrolyte imbalance in COVID-19 patients due mainly to a severe diarrhea.^(^^8^^)^

It should be noted that diarrhea has been described as one of the COVID-19 symptoms,^(^^9^^)^ which may have more negative effects on these patients. *Senna Makki* can also cause other side effects, including vomiting, stomach discomfort, nausea, cramps, and abdominal pain.^(^^8^^,^^10^^)^ Furthermore, its long-term use has been associated with heart function disorders, muscle weakness, liver damage, or even death in worse cases.^(^^10^^)^ In parallel to this, the effects of other remedies and medicines may be being erroneously assigned to *Senna Makki*.

All of these issues demand the implementation of comprehensive measures to raise awareness among the population, with special emphasis on the veracity of information made available by the media on possible treatments for COVID-19.

## RECOMMENDATIONS AND IMPLICATIONS ON THE USE OF MEDICINES FOR COVID-19

According to science principles, we strongly recommend that the indication/prescription of any medicines for COVID-19 should be based on solid evidence and/or aligned with guidelines of official international/national health organizations/societies.

The practice of self-medication with medicines in inappropriate doses or for populations with contraindications to their use may lead to serious consequences, and even death.^(^^11^^)^ Therefore, health professionals should always be on the lookout for updates on COVID-19 in the scientific literature, as well as on new regulations issued by scientific bodies and societies. Any empirical treatment at this time may result in imminent risks for any patient, and thus we should be aware and remember of this.

## CONCLUSION

Finally, given the current situation in Pakistan, we suggested that the population should adopt all preventive measures against the severe acute respiratory syndrome coronavirus 2 (SARS-CoV-2) infection, including wearing masks, cleaning hands with soap and water or alcohol-based hand rub, avoiding crowds, and trying to prefer well-ventilated spaces. In addition, all others restrictive measures for social distancing imposed by health authorities should be followed. We hope that SARS-CoV-2 vaccines will be soon available for a large part of the population, and may result in a significant improvement of the current scenario.
