# Supplementary material for: The media and the early dissemination of information on medicines for COVID-19: current scenario in Pakistan
Source: Einstein (Sao Paulo). 2021 Apr 8;19:eCE6354. doi: 10.31744/einstein_journal/2021CE6354 (PMC8029642; doi:10.31744/einstein_journal/2021CE6354)
Supplement: Translation

# Translation: As mídias e a divulgação precoce de informações sobre medicamentos para COVID-19: cenário atual no Paquistão

**Authors:** Marcos Roberto Tovani-Palone, Sajjad Ali

**Affiliations:** 1 Ribeirão PretoSP Brasil Faculdade de Medicina de Ribeirão Preto, Universidade de São Paulo, Ribeirão Preto, SP, Brasil.; 2 Karachi Pakistan Faculty of Medicine, Ziauddin University, Karachi, Pakistan.

Caro Editor,

Atualmente enfrentamos uma pandemia sem precedentes nos tempos modernos.^(^^1^^)^ Nesse contexto, as mídias podem exercer grande influência na população.^(^^2^^)^ Diante disso, existe uma preocupação com a propagação de possíveis benefícios de alguns medicamentos para a cura e prevençãoda doença pelo coronavírus 2019 (COVID-19), como a hidroxicloroquina, que têm sido precocemente divulgados por diversos meios de comunicação e governantes.^(^^3^^)^ Isso, por sua vez, pode resultar em impactos negativos na saúde da população.^(^^4^^)^ Neste artigo, nós apresentamos brevemente o cenário atual no Paquistão sobre a divulgação precoce do uso de medicamentos para COVID-19.

## CENÁRIO NO PAQUISTÃO

A pandemia da COVID-19 afetou a maioria dos países no mundo e também o Paquistão. Apesar disso, a população paquistanesa está menos vigilante, considerando que a adesão ao *lockdown* tem sido gradualmente menor, fato que tem contribuído para o grande aumento do número de casos confirmados da doença.^(^^5^^)^ Parece que até agora as pessoas ainda não perceberam que estamos enfrentando uma grave pandemia, a qual requer ações urgentes e disciplina. Além disso, alguns efeitos não comprovados de ervas naturais têm sido divulgados nas mídias e em *sites* da Internet como possíveis tratamentos para COVID-19, e muitos habitantes passaram a consumi-las. A erva mais popular no país é a *Cassia angustifolia Vahl*, conhecida como sene,^(^^6^^)^ que é geralmente utilizada como laxante.^(^^7^^)^ Contudo, essa erva pode ser uma ameaça à vida, ao invés de ser curativa. Isso é devido aos efeitos do chá de sene apresentarem potencial para causar desequilíbrio eletrolítico em pacientes com COVID-19, principalmente em decorrência de um quadro de diarreia grave.^(^^8^^)^

É importante mencionar que a diarreia tem sido descrita como um dos sintomas da COVID-19,^(^^9^^)^ o que pode acarretar mais problemas para esses pacientes. O sene também pode causar outros efeitos adversos, incluindo vômito, desconforto estomacal, náusea, cãibras e dor abdominal.^(^^8^^,^^10^^)^ Além disso, seu uso prolongado tem sido associado com distúrbios da função cardíaca, fraqueza muscular, lesão hepática e até óbito, em casos graves.^(^^10^^)^ Em paralelo, os efeitos de outros medicamentos podem ser erroneamente atribuídos ao uso do sene.

Logo, tais situações demandam a implementação de medidas abrangentes, a fim de melhorar a conscientização da população, em especial sobre a veracidade das informações disponibilizadas pelas mídias a respeito de possíveis tratamentos para COVID-19.

## RECOMENDAÇÕES E IMPLICAÇÕES DO USO DE MEDICAMENTOS PARA COVID-19

De acordo com princípios científicos, recomendamos fortemente que a indicação/prescrição de quaisquer me­di­camentos para COVID-19 seja baseada em evidências sólidas e/ou alinhadas às diretrizes de organizações/sociedades de saúde nacionais e internacionais.

A prática de automedicação em doses inapropriadas ou para população com contraindicações para seu uso pode levar a sérias consequências e até mesmo ao óbito.^(^^11^^)^ Profissionais de saúde devem estar sempre atentos a novidades em relação ao que é publicado na literatura científica sobre a COVID-19, bem como a respeito de novas regulamentações emitidas por órgãos e sociedades científicas. Qualquer tratamento empírico, neste momento, pode resultar em risco iminente para os pacientes. Desse modo, devemos estar cientes e nos lembrar disso.

## CONCLUSÃO

Considerando a situação atual no Paquistão, sugerimos que a população cumpra todas as medidas preventivas contra a infecção pelo coronavírus da síndrome respiratória aguda grave 2 (SARS-CoV-2), incluindo o uso de máscaras, a higienização das mãos com água e sabão ou álcool em gel, o afastamento de aglomerações e a preferência por ambientes bem ventilados. Além disso, todas as demais medidas restritivas de isolamento sociais impostas pelas autoridades de saúde devem ser seguidas. Esperamos que as vacinas contra o SARS-CoV-2 estejam disponíveis em breve para a maioria da população, resultando em uma melhora significativa do cenário atual.
